# Validation of the Moroccan arabic version of the low anterior resection syndrome score

**DOI:** 10.1186/s12876-020-01463-0

**Published:** 2020-10-13

**Authors:** Hajar Essangri, Mohammed Anass Majbar, Amine Benkabbou, Laila Amrani, Raouf Mohsine, Amine Souadka

**Affiliations:** Surgical Oncology Department, National Institute of Oncology, Ibn Sina University Hospital, Mohammed Vth University in Rabat, Rabat, Morocco

**Keywords:** Rectal neoplasms, Patient outcome assessment, Quality of life, Psychometrics, Postoperative complications, Low anterior resection syndrome

## Abstract

**Background:**

Sphincter sparing surgery is oftentimes associated with bowel dysfunction complaints, namely the low anterior resection syndrome (LARS). The LARS questionnaire is widely used to assess this syndrome. The aim of this observational study is to translate this tool into arabic and test its psychometric properties in rectal cancer patients, in order to ease its use in clinical practice and future research.

**Methods:**

The LARS questionnaire was translated to arabic and administered to a total of 143 patients. A subgroup of 42 patients took the test twice for test-retest reliability. Internal consistency was examined through cronbach’s alpha. The score results were correlated to the EORTC QLQ-C30 questionnaire for convergent validity assessment, while discriminant validity was established through the ability of the LARS score to differentiate patients with different clinical and pathological criteria.

**Results:**

The Moroccan Arabic version of the LARS score was completed by 143 patients. The internal consistency was demonstrated through a cronbach alpha score of 0.66. The agreement between the test and retest was established by a Bland Altman plot with 95% limits of agreement. 85.6% of patients remained in the same LARS category. The LARS score showed negative correlation with all five of the QLQ-C30 functional scales as well as positive correlation to the diarrhea symptom scale. The questionnaire score differed between patients according to their tumor location, chemoradiotherapy, type of mesorectal excision and anastomosis.

**Conclusion:**

The Moroccan Arabic version of the LARS score shows good psychometric properties and can be used for bowel dysfunction assessment in clinical and research settings.

## Background

With the evolving surgical management of rectal cancer and the impact of radiochemotherapy on tumor size and resectability, the number of sphincter sparing surgeries has increased as well as the rate of patients with bowel dysfunction [1–3]. This major complication ranges from partial and occasional to total incontinence, with increased frequency and urgency, or constipation and incomplete emptying, all encompassed in the low anterior resection syndrome (LARS) [4, 5].

This syndrome is associated with a negative impact on the quality of life [6], and is subject to a multitude of assessment tools most of which incorporate the same parameters, including the nature of incontinence (flatus, liquid seepage, liquid incontinence, solid incontinence), the incontinence type (active awareness, passive non-awareness, urge incontinence), the quantity of loss, the frequency of incontinence episodes, and accompanying complaints such as abdominal/pelvic pain and obstructed defecation. Among these assessment methods, the low anterior resection syndrome score (LARS score) has been specifically elaborated to explore the low anterior resection syndrome in patients after curative rectal surgery [7].

To date, a multitude of translation and validation studies have proven this tool to be both valid and reliable in different languages, countries and cultural contexts, yet the LARS score has never been translated into Arabic [8–17]. In Arabic speaking countries, colloquial Arabic varies between regions, with spoken versions being different from the Modern Standard Arabic and not always being mutually comprehensible [18]. The aim of this study is to translate the LARS score into Moroccan Arabic and to test its psychometric properties in Moroccan rectal cancer patients in order to allow its use as a low anterior resection syndrome assessment tool in clinical practice and future research.

## Methods

We conducted a transcultural validation of the Moroccan Arabic version of the LARS questionnaire in rectal cancer patients. This article is written following the STROBE (Strengthening the Reporting of Observational studies in Epidemiology) directive guidelines for observational studies [19].

### Data collection and participants

We retrospectively selected participating patients from the database of the National Oncology Institute of Rabat and the Private Oncology Center, on the period extending from January 2012 to March 2019. We did not include patients who underwent an abdominoperineal amputation, pseudocontinent perineal colostomy and those who had a stoma. The inclusion criteria were patients aged older than 18 years, diagnosed with rectal adenocarcinoma and who received low anterior resection with anastomosis creation or after a minimum 6 months interval after stoma reversal completed by October first 2019. Our exclusion criteria included cognitive dysfunction, the inability to speak Moroccan Arabic dialect and having a history of inflammatory bowel disease or any disease with bowel function impairment namely, Crohn’s disease, irritable bowel syndrome, ulcerative colitis or others.

Selected patients were approached during their check ups in the day clinic. Taking the high level of illiteracy and lack of other alternatives, patients who could not read received the help of an interviewer whose mission was to solemnly read the questions and answers to avoid bias. We also gave the questionnaire to patients over telephone interviews. Patients received the LARS questionnaire and the EORTC QLQ-C30 to prove convergent validity as well as the WEXNER score for the purpose of another study. Questionnaires with missing data were excluded from the analysis.

Written, informed consent was obtained from all patients who participated in the study at the time of their initial visit so that their clinical data could be used for clinical studies so long as their privacy was not jeopardized.

### Description of the instruments

The LARS was first developed on a nationwide cohort study of 961 Danish patients, then translated to English in 2012 [7, 9]. The LARS questionnaire consists of five items: “incontinence for flatus,” “incontinence for liquid stool,” “frequency of bowel movements,” “clustering of stools” and “urgency”. Each item has three to four response choices that are assigned with different score values. The third item has four choices, including “>7 times per day,” “4 to 7 times per day,” “1 to 3 times per day,” and “less than once per day,” assigned with values of 4, 2, 0, and 5 respectively. All the other four items have three choices, including “no, never,” “yes, less than once per week,” and “yes, at least once per week,” and are assigned with the values of 0, 4, and 7 for the first item; 0, 3, and 3 for the second item; 0, 9, and 11 for the fourth item; and 0, 11, and 16 for the fifth item, respectively.

The total score ranges from 0 to 42 and according to the results, three different groups are categorized as follows: 0 to 20 points - no LARS, 21 to 29 points - minor LARS and 30 to 42 points - Major LARS [7, 9].

### Translation process

We obtained permission from the original authors of the LARS [9, 20] in order to translate and use the questionnaire. The translation to Moroccan Arabic was performed by two independent professional translators who discussed the translations until a provisional consensual version was reached. Thereafter, a third translator who wasn’t familiar with the English version, back translated the agreed Arabic version to English. The three translators compared the English back-translated version against the original and a final Moroccan Arabic version was formed (Additional file 1). We conducted a preliminary test on a sample group from the target population to ensure the understanding and absence of difficulties answering the questions.

The translations aimed at achieving conceptual equivalence rather than a word-for-word translation and the process followed the recommendations of the WHO and the European Organisation for Research and Treatment of Cancer (EORTC) [21, 22]

### Psychometric validation

The psychometric validation consists of demonstrating the reliability of the instrument through testing its internal consistency and reproducibility as well as by examining the convergent and discriminant validity. This process is in compliance with the standards published by the American Educational Research Association [23].

#### Reliability

Reliability of the instrument was evaluated through Internal consistency and reproducibility, which we investigated by the means of cronbach’s alpha coefficient and test retest reliability respectively. A high positive value for Cronbach’s alpha superior to 0.70 suggests that the LARS score measures consistently.

We randomly selected a group of patients who responded to the questionnaire a second time after an interval of 2 to 4 weeks. Patients were asked if they had experienced any significant change in bowel function between the first and the second test and those confirming a change in bowel function were excluded from the test– retest analysis. The correlation between the numerical value of the LARS score at the first and second test was assessed by the means of a Bland–Altman plot with 95% limits of agreement, as well as through measuring the intraclass correlation coefficient, which is considered to be adequate when superior to 0.80. Furthermore, for each of the 5 individual questions of the score, the agreement between the first and second response was explored by means of computing the percentage of perfect, moderate, and no agreement. A perfect agreement was assigned when participants ticked off exactly the same category at the first and second test, moderate agreement was assigned when responses differed by only 1 category, and no agreement was assigned when responses differed by 2 or 3 categories at the 2 tests.

#### Validity

For the purpose of convergent validity testing, the EORTC QLQ-C30 questionnaire was used. This quality of life assessment tool includes five functional subscales (i.e., physical functioning, role functioning, emotional functioning, cognitive functioning, and social functioning), three symptom subscales (i.e., fatigue, nausea and vomiting, and pain), a global QoL subscale, and six single symptom items (i.e., dyspnea, insomnia, appetite loss, constipation, diarrhea, and financial difficulties). We specifically analysed the correlation between the LARS score results and both the functional scales and the diarrhea symptom scale [24].

With regards to the scoring instructions for this instrument, a high score represented a high QoL or a high level of functioning for the global QoL subscale and functional subscale. Opposingly, for a symptom subscale/item the higher the score, the more severe the symptoms are [25].

For discriminant validity testing, we primarily hypothesized that the LARS score would differentiate the bowel functions of patients with different demographic or clinical features such as sex, age, length of postoperative period (time since stoma-free rectal resection surgery or reversal surgery of temporary stoma), distance of the tumor from the anal verge, radiation therapy, extent of mesorectal excision, prior temporary stoma and post operative complications.

### Statistical analysis

Demographic and clinical variables were analyzed by using descriptive statistics. When assessing the test-retest reliability, the Spearman correlation coefficient was used because both scores at the first and second surveys are non-normally distributed. The correlations between the LARS score and the subscales of the EORTC QLQ were evaluated by using Spearman’s correlations as well. All *p* values < 0.05 were considered statistically significant. All statistical analyses were performed using IBM SPSS Statistics (version 22).

### Literature review

We conducted a comprehensive search of prior translations and validations of the LARS score in MEDLINE (PUBMED) on a search period extending from January 1st, 2012 to October 1st, 2019. The MeSH and main keywords were the following: “Rectal neoplasms”, “Postoperative complications”, “fecal incontinence”, “Patient Outcome Assessment”, “Surveys and Questionnaires”, “Translations”, “Reproducibility of Results”, and “Psychometrics”.

### Ethics

The study received the approval of the biomedical ethics committee of the faculty of medicine in Rabat (Number: 99/19) and was approved and published in the international clinical trials database under the title: Validation of the Moroccan Arabic Version of the Low Anterior Resection Syndrome (LARS) and Wexner Score of Continence Among Rectal Cancer Patients (MA_LARSWEX) and the number: NCT04128657 [26].

## Results

**Fig. 1:**
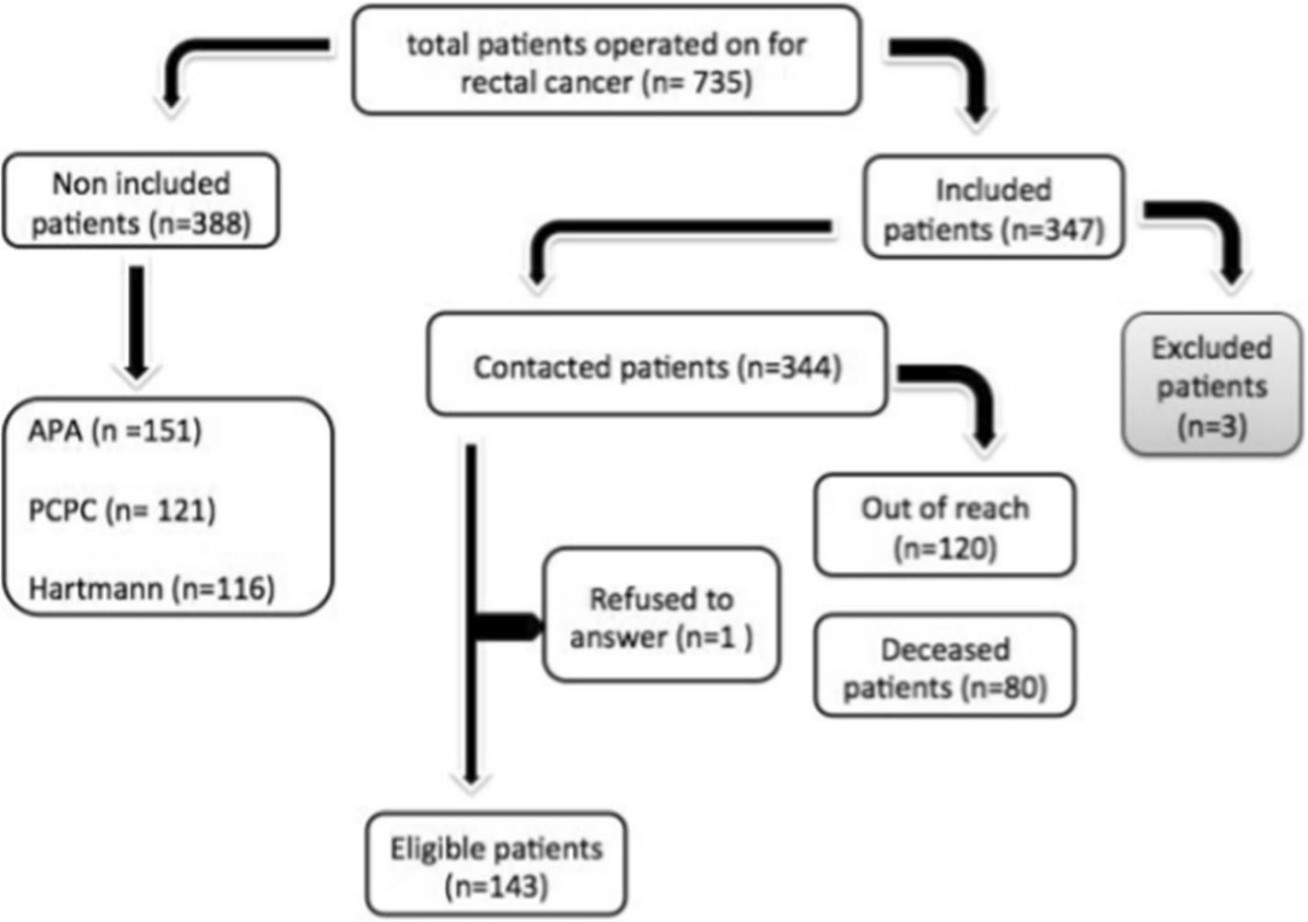
Patient selection flowchart

In the period from January 2012 to March 2019, we identified a total of 735 patients operated on for rectal neoplasms, among which 143 patients were deemed eligible according to our inclusion criteria and responded to the questionnaire with a response rate of 99% (Fig. 1). Owing to the high level of illiteracy and geographical challenges, 44 patients received the help of an interviewer, while 7 were interviewed over the phone. Only 1 patient refused to respond to the measurement tool. The mean interview time was 7 min.

### Patients clinical and demographic characteristics

Among the 143 responders, 66(46.2%) were female. The mean age of respondents was 58.15 ± 13.23 years, with 16.8% of patients younger than 45 years. We categorized tumor location into upper, middle and lower rectum. 59.4% of our patients received neoadjuvant chemoradiotherapy and 63.6% of patients underwent adjuvant chemotherapy. As regards surgery, we differentiated between patients with colorectal and coloanal anastomosis, as well as those who benefited from either partial or total mesorectal excision. We also identified patients with no LARS, minor LARS or major LARS. Further clinical and demographic characteristics are shown in Table 1.

**Table 1:** Clinical and demographic characteristics of patients

Variables	Description
Mean follow up time	37,25 months
Age (years)	
● Mean age	58.15 ± 13.23
● < 45 years	24(16,8%)
● > 45 years	117(81,8%)
● Unavailable	2 (1,4%)
Sexe	
● Female	66 (46.2%)
● Male	77 (53.8%)
Tumor location (cm)	
● Upper rectum (10–15)	58 (40.6%)
● Middle rectum (5–10)	61 (42.7%)
● Low rectum (0–5)	24(16.8%)
Neoadjuvant chemoradiotherapy	
● No	85 (59.4%)
● Yes	58 (40.6%)
Anastomosis type	
● Colorectal	115 (80.4%)
● Coloanal	28(19.6%)
Type of mesorectal excision	
● Partial	70 (49%)
● Total	73(51%)
Adjuvant chemotherapy	
● Yes	91 (63.6%)
● No	52(36.4%)

### Reliability

The internal consistency of the LARS score in the 143 patients assessed through cronbach’s alpha was found to be at 0,66.

**Fig. 2:**
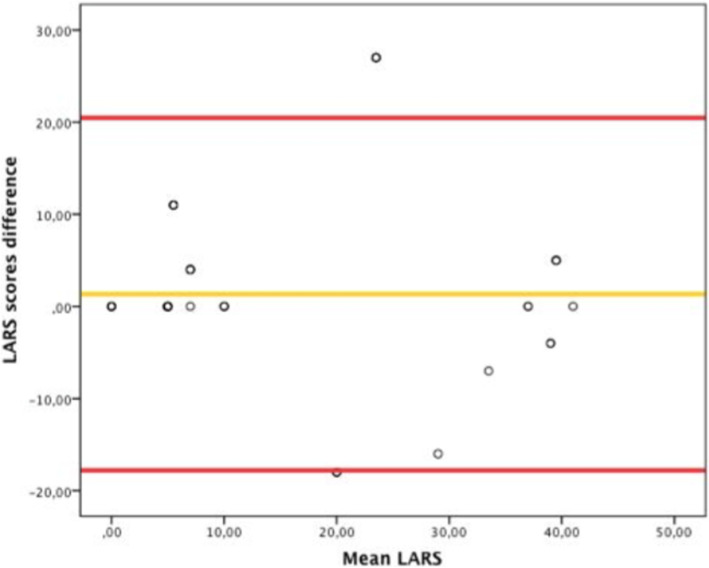
Bland–Altman plot with 95% limits of agreement illustrating the difference between LARS scores at the first and second test

The test- retest study was applied on 42 patients with a median period between both tests of 2 weeks. The differences between the first and second numerical values of both LARS tests are established by the means of a Bland Altman plot with 95% limits of agreement (Fig. 2). The degree of agreement between the initial test and retest for each of the five LARS score items and the LARS category (no, minor, major LARS) is presented in Table 2. The interclass correlation showed good reliability (ICC = 0.88).

**Table 2:** Agreement levels of the LARS categories and score items between the test and retest

	Agreement level		
	Perfect (%)	Moderate (%)	None (%)
LARS category	85.6%	7.1%	7.1%
Item 1	90.4%	7.1%	2.3%
Item 2	100%	0 (0)	0 (0)
Item 3	88%	7.1%	4.7%
Item 4	85.6%	7.1%	7.1%
Item 5	76.2%	7.1%	16.6%

### Validity

The LARS score showed statistically significant negative correlation with each one of the five EORTC QLQ-C30 functional scales. Positive correlation has been demonstrated as well as with the diarrhea symptom scale Table 3.

**Table 3:** Convergent validity of the LARS score according to the QLQ C30 score scales

Dimensions of QLQ-C3o	Total score of the LARS	
	R value	***P*** value
Global QLQ	-0,322	< 0,001
Physical functioning	-0,160	0,05
Role functioning	-0,190	0,023
Emotional functioning	-0,242	0,004
Cognitive functioning	-0,242	0,004
Social functioning	-0,242	0,004
Diarrhea	0,516	< 0,001

**Fig. 3:**
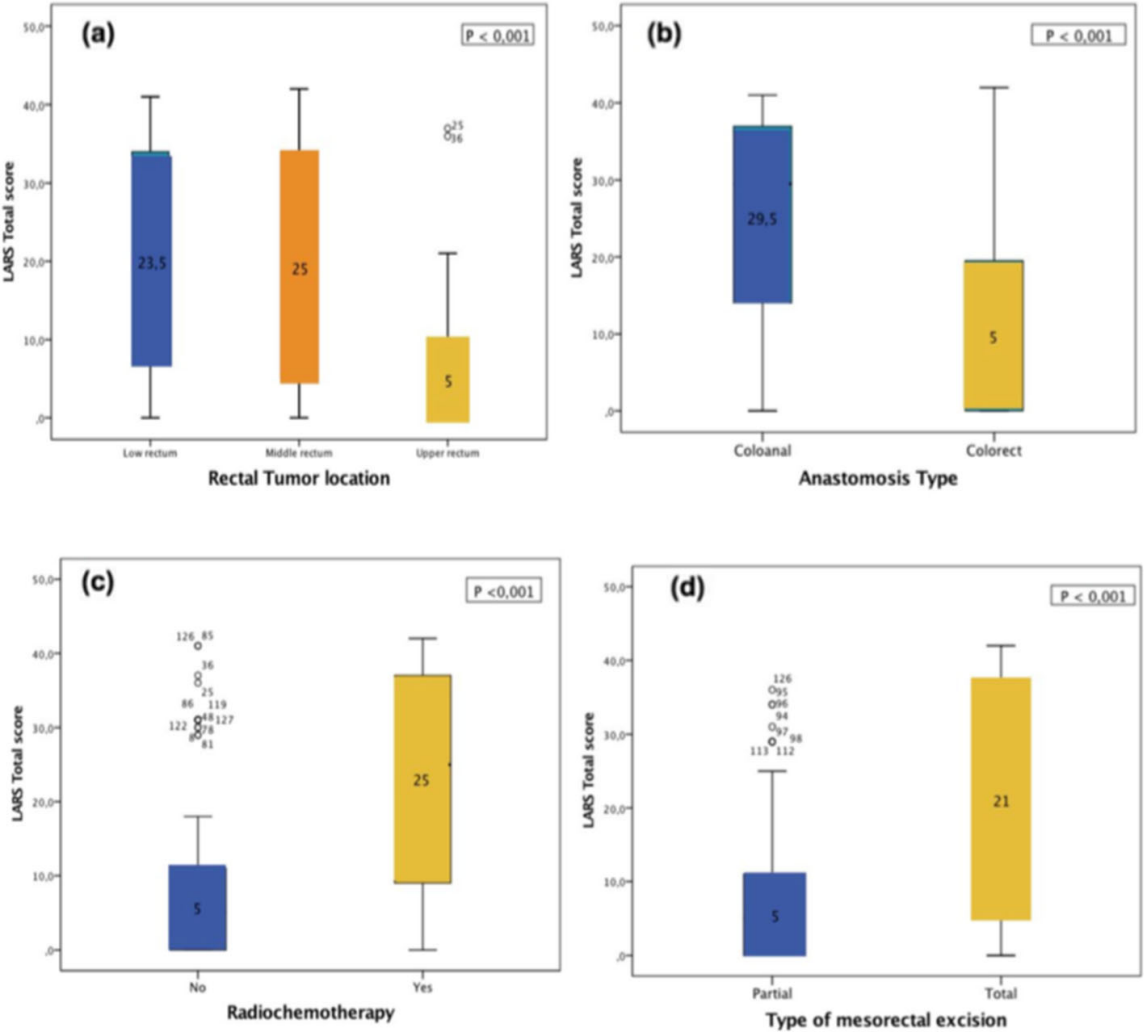
Boxplots illustrating the LARS total score according to: (**a)** rectal tumor location, (**b)** anastomosis type, **(c)** radiochemotherapy administration or not, **(d)** type of mesorectal excision

The LARS score was able to detect differences based on rectal tumor location (*p* < 0,001), coloanal or colorectal anastomosis (*P* < 0,001), type of mesorectal excision (P < 0,001) and patients who had/had not undergone radiotherapy (P < 0,001) (Fig. 3).

We conducted a search of all previously validated versions of the LARS questionnaire. This latter has been translated into 24 languages among which 14 validation studies have already been published whilst other versions are still in the process of validation (Appendix 1).

## Discussion

This study has shown that the Moroccan Arabic version of the LARS score has good psychometric properties when applied to rectal cancer patients. Although the LARS questionnaire and EORTC QLQ-C30 are relatively long, the response rate was 99% for a mean interview time of 7 min which shows good acceptability and understanding of the questions.

The mean follow-up time was 55.5 months in the original LARS score development research [7]. Other subsequent validation studies included relatively shorter periods such as the Lithuanian and Chinese [10, 12]. In our study, the mean follow up period was 37,25 months in order to allow sufficient time for bowel function stabilization.

The internal consistency measurement through cronbach’s alpha coefficient, yielded in a coefficient slightly lower than the acceptable level of 0,70. In fact the cronbach’s alpha has been subject to some criticism as to the relationship between longer scales and higher alpha coefficients [27].This could be an explanation to our findings as the LARS questionnaire includes only 5 items, particularly in the shortage of cronbach’s alpha use in the previous international validations of this questionnaire.

We determined the test retest reliability of the score by administering the questionnaire for a second time to a group of 42 patients after an interval of 2 weeks, which demonstrated good reliability (ICC = 0,88) and proportions of moderate and high agreement exceeding 85% compared to only a few patients showing no agreement, which was the case in many other international studies.

A thorough screening resulted in a total of 14 validation studies of the low anterior resection syndrome questionnaire. In these previous validations, convergent validity testing was either proven through correlating the LARS results to specific fecal incontinence measurement tools, namely the wexner score, or to quality of life assessment questionnaires such as the EORTC QLQ C29 and the EORTC QLQ C30.

The chinese version illustrated the correlation between the EORTC QLQ-C30 and the LARS score, showing statistically significant correlation except for the cognitive functioning scale and the nausea and vomiting symptom scales. Similarly, we tested the convergent validity with the EORTC QLQ-C30 functional scales and diarrhea symptom scale, as the latter represents the most relevant symptom to the low anterior resection syndrome. Significant correlation with all functional scales and the diarrhea symptom scale was established.

The original Danish validation as well as some other translations, on the other hand, correlated the score with only one additional quality of life related question: “Overall, how much is your QoL influenced by your bowel dysfunction?,” with four response options: “not at all,” “a little,” “some,” or “a lot.” [8, 9, 11, 13]

The discriminative validity of the arabic LARS score was equivalent to previous studies, with statistically significant differences according to tumor location, type of mesorectal excision, coloanal or colorectal anastomosis and administration or not of radiotherapy. As to age and sex, no significant difference has been accordingly proven. Opposingly, previous studies perceived female patients aged 50 to 79 to have a higher proportion of Major LARS and worse functional outcome, while age remains a subject of controversy between studies correlating young age to worse LARS scores and those not finding a significant correlation [28–31].

Postoperative bowel dysfunction is a major health issue and its burden on patients quality of life is sometimes underestimated [32]. In Arabic communities, such manifestations may also have a strong cultural component which not only increases the quality of life impediment [33], but also represents a hurdle to adequate care due to shame and miscommunication [34]. Accordingly, the self-administered LARS questionnaire may help bridge the gap between cultural taboos and patient care. In addition, the use of the validated LARS score in daily practice will allow an objective assessment of bowel dysfunction prevalence following colorectal surgery and the severity of the quality of life impact in order to provide adequate treatment and follow up [35].

Our study has some limitations such as the retrospective observational aspect when selecting the patients. Taking the high level of illiteracy and lack of alternatives, it was not possible for the patients to complete the questionnaire, but rather with the help of an interviewer who was reading the questions or through phone communications. The number of patients involved was also a limitation.

## Conclusion

The Moroccan Arabic version of the LARS score has good psychometric properties and can therefore be used for bowel function evaluation in colorectal cancer patients in Morocco. The validation of a modern standard Arabic version of the LARS questionnaire could allow a more universal assessment in other Arabic speaking countries, however, the understanding of a standard Arabic version would be bound by the level of literacy. Broadly speaking, the universal assessment of the low anterior resection syndrome will allow the identification of patients in need of further care and ease the elaboration of universal solutions for bowel dysfunction and quality of life impairment in colorectal cancer patients.

### Supplementary information

**Additional file 1:.** Arabic version of the LARS questionnaire

## Data Availability

Derived data supporting the findings of this study are available from the corresponding author [AS] upon reasonable request.

## Appendix 1

**Table 4:** Literature review summary of the validated LARS score studies

Language of validation	Number of patients	Correlated tools of convergent validation testing
Dutch [8]	165 patients	1 quality of life question
German [9]	801 patients	1 quality of life question
Spanish [9]		
Swedish [9]		
Danish [9]		
Chinese [10]	102 patients	EORTC QLQ-C30 questionnaire and EORTC QLQ-C29 questionnaire.
Japanese [11]	136 patients	1 quality of life question
Lithuanian [12]	111 patients	wexner score
Slovenian [13]	100 patients	1 quality of life question
Portugese [14]	154 patients	EORTC QLQ-C30
Russian [15]	-	-
Greek [16]	112 patients	EORTC QLQ-C30 questionnaire and EORTC QLQ-C29 questionnaire
English [17]	451 patients	EORTC QLQ-C30 questionnaire
Norwegian [19]	961 patients	-

## Abbreviations

LARS: Low anterior resection syndrome
EORTC: European Organisation for Research and Treatment of Cancer
QLQ-C30: Core Quality of life questionnaire version 3
QoL: Quality of life
