# Supplementary material for: Validation of the Moroccan arabic version of the low anterior resection syndrome score
Source: BMC Gastroenterol. 2020 Oct 13;20:333. doi: 10.1186/s12876-020-01463-0 (PMC7552529; doi:10.1186/s12876-020-01463-0)
Supplement: Supplementary file 1 — Additional file 1:. Arabic version of the LARS questionnaire [file 12876_2020_1463_MOESM1_ESM.docx]

**Appendix 1 : Arabic version of the LARS questionnaire**

1. **واش كيوقع ليك شي مرات لي كتقدرش تتحكم في النفس؟**

- لا، حتا مرة.
- أيه، قل من مرة وحدة في الأسبوع.
- أيه، على الأقل مرة في الأسبوع.

1. **واش كيوقع ليك شي مرات لي كيفلت ليك الخروج جاري؟**

- لا، حتا مرة.
- أيه، قل من مرة وحدة في الأسبوع.
- أيه، على الأقل مرة في الأسبوع.

1. **شحال من مرة كتدير الخروج؟**

- اكثر من سبعة ديال المرات في اليوم.
- ما بين ٤ حتا ل ٧ ديال المرات في اليوم.
- ما بين مرة حتال ٣ ديال المرات في اليوم.
- اقل من مرة وحدة في اليوم.

1. **واش شي مرات خاصك تدير الخروج ديالك على الأقل ٢ مرات في الساعة؟**

- لا، حتا مرة.
- أيه، قل من مرة وحدة في الأسبوع.
- أيه، على الأقل مرة في الأسبوع.

1. **واش شي مرات كتزير فالخروج ديالك لدرجة انك خاصك تجري للمرحاض؟**

- لا، حتا مرة.
- أيه، قل من مرة وحدة في الأسبوع.
- أيه، على الأقل مرة في الأسبوع.
